# Supplementary material for: Are carbon emissions trading and green financial instruments synergistic? -Comprehensive quantitative research based on content analysis
Source: PLoS One. 2024 Mar 7;19(3):e0298601. doi: 10.1371/journal.pone.0298601 (PMC10919601; doi:10.1371/journal.pone.0298601)
Supplement: S1 Data — (ZIP) [file pone.0298601.s001.zip › Data/地级市Z_F工作报告.zip!/░▓╗╒/╦▐╓▌╩╨/└┤╫╘╣1⁄2╓┌║┼í╛┬φ┐╦╩2╛▌═°í┐_2013.docx]

2013年1月7日在宿州市第四届人民代表大会第一次会议上

市长  张曙光

各位代表：

现在，我代表市人民政府，向大会报告政府工作，请予审议，并请市政协委员和其他列席人员提出意见。

一、加速崛起的奋进历程

市三届人大一次会议以来的四年，是我市经济持续发展、城乡统筹协调、民生不断改善、文化日益繁荣、社会和谐稳定的四年。全市上下在市委的坚强领导下，深入贯彻党的十七大、十八大和省第九次党代会、市第四次党代会精神，牢牢把握主题主线，齐心协力、苦干实干，圆满完成“十一五”目标，开启了加速崛起的新征程。

——四年来，我们始终坚持发展至上，着力优化经济结构，综合实力显著增强。初步核算，全市生产总值连跨五个百亿元台阶，由483.3亿元增至910亿元，年均增长12.9%；财政收入连跨六个十亿元台阶，由27.4亿元增至83亿元，年均增长32%；三次产业结构由31.7:31.4:36.9调至26.4:41.2:32.4。工业主导作用显著增强，规模以上工业增加值由116亿元增至248亿元，年均增长24.3%，净增产值超亿元企业129家。新认定高新技术企业10户，高新技术产业产值年均增长51.3%。创建中国驰名商标1件，省著名商标38件。农村经济稳步发展，国家级现代农业示范区、全国农村改革试验区获批，为全省唯一。粮食生产实现“九连增”，桥区跻身全国粮食生产先进单位。砀山成为国家级出口果蔬质量安全示范区。萧县荣获中国面粉加工强县称号。规模养殖比重达61%。农产品加工产值由259亿元增至600亿元，农业产业化综合水平居全省第一。市级以上龙头企业达329家，农民专业合作组织3500个，国家级示范社3个。林业总产值突破200亿元，桥、砀山位居全省林产业十强。粮食生产基本实现机械化。服务业发展提质增效，增加值年均增长11.6%，社会消费品零售总额由141.3亿元增至267亿元，年均增长17.3%，家乐福、合百大等企业相继入驻，“万村千乡”工程实现全覆盖。文化旅游、服务外包等现代服务业不断壮大。县域经济发展加速，萧县、泗县跻身省县域经济科学发展先进县，35个乡镇工业增加值超亿元，25个乡镇财政收入超千万元。

——四年来，我们始终坚持统筹协调，着力加大有效投入，城乡面貌明显改观。累计完成固定资产投资1780亿元，年均增长30%，开工建设亿元以上项目332个，“5161”项目库储备规模达1.3万亿元。京沪高铁、泗许高速一期建成通车，宿淮铁路、徐明高速、泗许高速二期、济祁高速加快建设，高速公路建成通车里程达213公里。“数字宿州”、“无线宿州”建设步伐加快，广播电视综合覆盖率分别达92.2%、93.8%。建成220千伏变电站5座、110千伏变电站6座。全力推进城镇扩容，城镇化率达34.6%。新一轮城市总体规划获批。市区累计投资400亿元，实施重点工程600项，新建、续建道路160公里，着手实施新汴河城区段治理、清水工程，人民路、拂晓大道、洪河路、淮河路跨铁路立交桥、三角洲公园等建成使用，大外环部分路段实现通车。加强城市管理，实施“五化”工程，新增绿量550万平方米。完成市区出租车更新换代。统筹推进县城和特色镇建设，灵璧、泗县、砀山政务新区和萧县凤城新区基本建成。15个乡镇被列入新农村建设示范镇。农田水利建设三年恢复性工程顺利完成，三年提升工程加快实施，五个县区均为全国小农水重点县，灵璧跻身全国农田水利建设先进县。美好乡村建设扎实推进，“村村通”道路工程完成5082公里，完成农村清洁工程59个、饮水工程286处、危房改造2.3万户、环境连片整治10个村，创建省级生态镇3个、生态村23个。

——四年来，我们始终坚持改革开放，着力承接产业转移，发展活力不断彰显。人事制度、公共财政、医药卫生、文化体制及农村综合改革稳妥推进。市演艺集团组建完成，“营改增”试点顺利实施，县级公立医院改革全面启动。农村金融产品和服务方式创新试点市工作扎实开展，徽商银行、淮海村镇银行入驻，灵璧农商行获准筹建，本富村镇银行、皖兴产业创投中心挂牌，4家企业进入上市辅导。新组建交投、工投、教投等多个融资平台，新发展融资性担保公司10家、保险公司17家、小额贷款公司7家，金融生态县创建活动扎实有效，四年累计存贷款实现翻一番，贷款余额增长2.3倍。积极承接产业转移，利用外资年均增长48%，累计到位内资超1600亿元，引资总量、增幅均位居全省前列；成功举办灵璧石文化节、黄淮海农资博览会、梨花旅游节、泗州戏艺术节、国际防腐蚀大会等节庆会展；发展环境持续优化，灵璧、萧县被徽商协会评为最佳投资区域，桥、泗县入选苏商最具投资价值城市。对外开放向纵深拓展，海关、国检开展正常业务，进出口总额年均增长46.9%。各类开发园区发展势头迅猛，高新技术产业开发区、中国现代制鞋产业城、纺织服装产业城、中国宿州（深圳）家居产业园高起点建设，宿马现代产业园区启动迅速，各县区开发区、22个新型乡村工业园、30个农民工返乡创业园呈现旺盛活力。非公有制经济蓬勃发展，新增民营企业3844家、个体工商户3.8万户。

——四年来，我们始终坚持以人为本，着力改善民计民生，社会保障更加有力。城镇居民人均可支配收入由11899元增至2万元，年均增长13.9%，农民人均纯收入由3671元增至6578元，年均增长15.7%。各类民生支出335亿元，占财政支出比重80%以上。民生工程投入资金140.4亿元，工作成效跻身全省先进。连续两年共实施19件惠民实事。新增城镇就业16.9万人，城镇登记失业率控制在4.5%以内。着力提高社会保障水平，8.9万被征地农民实现应保尽保，“五险”参保人数达到169.8万人次，城乡居民养老保险试点、最低生活保障制度实现全覆盖。高度关注特殊群体，发放城乡居民低保金11.5亿元，企业退休人员养老金、农村五保户供养标准逐年提高，新建、改扩建敬老院157个，社会福利中心、残疾人康复中心基本建成。开工建设各类保障性住房9万余套（间），廉租房配租5000余套（户）。农民工技能培训达15万人，完成200个村的扶贫开发任务。

——四年来，我们始终坚持和谐发展，着力创新社会管理，社会大局保持稳定。大力推进科技创新，组织实施国家和省科技项目108项，申请专利2256项，市及桥、萧县、砀山、灵璧通过全国科技进步考核。坚持教育优先发展，投入15亿元，实现城乡同步免费义务教育，受益学生300多万人次。泗州戏学院开始招生，宿州职业技术学院通过人才培养评估验收。中小学布局加速调整，教育园区启动建设，皖北卫生职业学院即将获批。医疗卫生服务体系进一步完善，皖煤总医院、矿建总医院实施属地管理，市立医院新院区开工，三家县级医院实施整体搬迁。新农合参合率达97.6%。重大传染病防治取得明显成效，荣获全国无偿献血先进市、全国白内障无障碍市称号。文化事业和文化产业快速发展，文艺精品创作硕果累累。《渔鼓道情》等7个项目入选国家级非物质文化遗产保护名录。博物馆、民间艺术馆建成开放，文化艺术中心主体完工，各县文化产业园区建设加快，农家书屋、文化站实现全覆盖。萧县、砀山入选中国民间艺术之乡，灵璧被命名为中国十大文化特色旅游名县、中国观赏石综合基地。群众性精神文明创建活动广泛开展，涌现出最美孕妇、全国优秀教师等先进典型。竞技体育和群众性体育活动双向推进，全民健身中心开放使用，体育馆建设加快，成功举办国际女子网球巡回赛。低生育水平持续稳定，连续三年在全省考核中取得优异成绩。生态建设成效明显，森林覆盖率达27.9%，城区污水处理能力达8万吨，节能减排完成省控目标。持续加大安全生产和食品药品监管力度，应急管理体系不断完善，市场价格调控积极有效。深入推进“书记带头大走访”，信访秩序持续好转。平安宿州建设扎实推进，人民群众安全感稳步上升。“五五”普法通过验收，“六五”普法启动实施。实现全国“双拥模范城”六连冠。民族宗教、外事侨务、对台、档案、地方志工作得到加强，科协、气象、防震减灾工作扎实有效，妇女、儿童、老龄、红十字、残疾人事业实现新发展。

——四年来，我们高度重视政府自身建设，着力强化执政能力，行政效能大幅提升。大力推进依法行政，行政决策机制不断完善。注意听取各方意见和建议，自觉接受人大法律监督、政协民主监督和社会舆论监督，按期办结人大议案13件、代表建议360件、政协委员提案1074件。加强与工会、共青团、妇联等群众团体的联系。深入开展以“六要六不要”为中心内容的“正风气、提效能”活动，深化行政审批制度改革，实施行政服务“四个一”工程，政府工作执行力大幅提升。实行领导干部工作计划、每日工作动态网上公开，畅通政风行风热线，设立网络发言人，网络问政日趋完善。认真落实廉政建设责任制，支持监察、审计部门依法履行监督职责，大力建设节约型机关，政风建设取得新成效。

各位代表！

本届政府任期的四年，正值收官“十一五”、实施“十二五”的关键阶段。进入“十二五”的两年来，我们始终不渝高举发展大旗，牢牢把握主题主线，着力推进“双轮驱动、三化同步”，不动摇、不懈怠、不折腾，引领广大干群思想同心、目标同向、行动同步，全市上下形成了思发展、议发展、心无旁骛抓发展的浓厚氛围。两年来，我们始终不渝抓主抓重、争先争优，紧盯“动态指标进全省前八”目标不放松，抓投入保增长，抓三农稳基础，抓城建筑平台，抓民生促和谐，抓效能优环境，大力开展园区和城乡建设突破年、社会管理创新年、机关效能建设年等活动，深入实施项目“5161”、工业“7233”、农业“4168”等工程，通过重点突破，推动全局工作开展。两年来，我们始终不渝提速提效、苦干实干，大力倡导“五加二”、“白加黑”的工作作风，强化责任分解，密集督查调度，严格考核奖惩，激励先进，鞭策后进。市政府领导班子以身作则，率先垂范；各级各部门主动作为，真抓实干；广大公务人员勇于担当，甘于奉献；各行各业积极参与，力争上游，有力促进了经济社会持续快速发展。2011年，荣获省政府目标管理考核先进单位，综合排名居全省第六，实现了历史性突破。2012年，各项经济指标增幅继续保持全省先进位次，预计生产总值增长12.5%，财政收入增长29.6%，固定资产投资增长25%，规模以上工业增加值增长18%，城乡居民人均收入均增长15%，在经济下行压力不断加大的形势下，实现了整体经济的逆势上扬。

各位代表！

我们共同走过坚实的四年，此时此刻，我们更加深切体会到，市委坚强领导，几大班子精诚团结，全市上下同心同德，是我们战胜困难夺取胜利的根本保证；坚持科学发展，推动“双轮驱动、三化同步”，是我们洼地起跳、弯道超车的必由之路；坚持解放思想，点燃工作激情，永不满足、永不懈怠，是我们直面挑战抢抓机遇的重要法宝；坚持发展为了人民，着力改善和保障民生，是我们一切工作的最终目的。这些经验，来自实践，弥足珍贵，必须始终坚持、不断发展。此时此刻，我们更加深切体会到，来之不易的发展成绩，得益于省委、省政府的坚强领导，得益于历届班子打下的坚实基础，得益于市人大、市政协和社会各界的监督支持，得益于全市上下干事创业的奋发努力。在此，我谨代表市政府，向奋战在各行各业的全体劳动者，向人大代表、政协委员、各民主党派、工商联、人民团体，向离退休老同志，向中央和省驻宿单位、人民解放军驻宿部队、武警官兵、公安干警，向参与宿州建设的海内外客商、广大援宿干部，以及所有支持宿州发展的各界人士，表示衷心的感谢，并致以崇高的敬意！

各位代表！

成就令人振奋，问题必须正视。毋庸置疑，在全省发展格局中，人口总量靠前，经济总量居中，人均水平靠后，是我们最大的市情。产业结构不优，新兴产业不足，财政保障能力不强，人民群众收入不高，中心城市功能不够完善，城乡发展不够平衡，县域经济发展滞后，是我们最大的实际。一些干部学习意识不强，思想解放不够，缺乏激情和锐气，缺乏责任和担当，不作为、乱作为，甚至消极腐败现象还不同程度存在，是我们最大的隐忧。对此，我们务必要保持清醒头脑，切实采取有效措施，努力加以解决。

二、未来五年的发展愿景

未来五年，是我市全面建成小康社会的关键时期。综观新阶段新形势，我市仍处在大有可为的黄金发展期。党的十八大为我们指明了前行方向，注入了新的动力，跻身中原经济区规划，振兴皖北战略深入实施，多层政策优势叠加，发展条件不断改善。多年持续高强度投入，发展能量加速释放，广大干群干事创业的激情正在迸发，加速崛起基础坚实。尽管前进道路上还有许多风险挑战，但发展机遇前所未有。我们一定要进一步增强发展自信，以更高的站位规划未来蓝图，以更大的气魄推动改革发展，奋力谱写美丽宿州新篇章。

今后五年工作的总体要求是：高举中国特色社会主义伟大旗帜，以邓小平理论、“三个代表”重要思想、科学发展观为指导，深入贯彻党的十八大精神，紧紧围绕主题主线，坚持双轮驱动，实施三大战略，推进四化同步，在皖北振兴中勇当先锋，在全省发展中争先进位，在中部崛起中奋发有为，努力建设实力宿州、活力宿州、和谐宿州、幸福宿州，为全面建成小康社会打下具有决定性意义的基础。

奋斗目标是：围绕“总量争先进位、人均缩小差距、收入赶上全省”的总体要求，力争实现生产总值、财政收入、固定资产投资、战略性新兴产业和服务业增加值、城乡居民人均收入“七个翻番”。生产总值冲刺2000亿元，财政收入突破200亿元，固定资产投资五年累计突破5000亿元。县域经济实现“增速超全省、财政增两倍”。

——构建现代产业体系新优势，产业结构更加优化，竞争实力更加突出。坚持走新型工业化道路，推进工业化、信息化深度融合，做大做强传统产业，着力突破战略性新兴产业，构建更具竞争力的现代产业体系。加快培育2个超千亿元的支柱产业，规模以上工业企业达1800户，工业增加值力争达1000亿元。以“两区”建设为平台，提升现代农业发展，推动农业产业化、标准化、集约化、信息化发展。加快发展金融、信息、服务外包、现代物流、文化旅游等产业，服务业增加值达700亿元，二、三产业增加值占生产总值比重提高到85%左右。

——开创城乡统筹发展新局面，城市品质更加优秀，城乡发展更加协调。以新型城镇化为引领，统筹推进城区、产业园区、新型农村社区“三区”建设，加快构建布局合理、规模适度、功能互补、城乡互动的城镇体系，城镇化率达到49%以上。主城区实现“双百”目标。2个以上县城建成中等城市，一批特色城镇加快崛起，50%以上的中心村按美好乡村标准建设。全面加快高速公路、高速铁路、内河航道、快速通道等基础设施建设，积极申报宿州机场项目，强化综合交通枢纽地位。提高城市综合实力，争创国家和省级园林、卫生、文明城市。

——展现改革开放新气象，发展活力更加强劲，发展环境更加优越。加快重点领域和关键环节的改革步伐，着力构建充满活力、富有效率、科学发展的体制机制。加快承接产业转移，创建国家级开发区，到2017年，招商引资总量达到7000亿元，利用外资质量和水平全面提升。高度重视人才引进培养使用，创新创业氛围更加浓厚，要素分配和激励机制更加完善，公平竞争格局进一步形成。行政效能显著提高，发展环境不断优化，各类市场主体活力充分释放。

——迈出文化强市建设新步伐，文化发展更加繁荣，社会风尚更加良好。大力弘扬时代精神，不断拓展公民道德实践和精神文明创建活动，社会主义核心价值观深入人心，良好思想道德风尚进一步弘扬，全民文明素质显著提高。文化事业全面发展，公共文化服务体系不断完善，文化产业快速推进，增加值占生产总值比重达到5%以上。城市文化影响力显著增强，成为内聚合力、外具魅力、特色鲜明的区域文化中心。

——实现生态文明建设新跨越，生态特色更加鲜明，环境质量更加优良。牢固树立生态文明理念，资源节约型、环境友好型的产业结构、增长方式和消费模式加快形成。积极发展循环经济，耕地保护制度、节约用地制度、水资源管理制度建立健全，完成节能减排省控目标，城镇污水处理率和生活垃圾无害化处理率均达到85%，农村生活垃圾处理率达到45%，森林覆盖率达到30.5%以上。

——提升保障改善民生新水平，社会管理更加有序，人民生活更加幸福。坚持居民收入增长和经济发展同步，劳动报酬增长和劳动生产率提高同步。基本公共服务均等化基本实现，全民受教育程度明显提高，就业更加充分，社会保障体系逐步完善，社会化养老服务水平明显提升，人人享有基本医疗卫生服务，住房保障体系基本形成。平安宿州建设成果不断巩固，社会管理创新更趋完善，群众合法权益得到切实保障，人民群众安全感显著增强，生活更加殷实、更有尊严、更加幸福。

三、开局之年的各项工作

今年是全面贯彻党的十八大精神的起步之年，是新一届政府的开局之年。综合考虑，经济社会发展主要目标是：经济总量超千亿，财政收入超百亿；生产总值增长12%以上，财政收入增长15%以上，固定资产投资增长20%以上，社会消费品零售总额增长15%以上，城乡居民人均收入均增长15%以上，城镇登记失业率控制在4.5%以内，人口出生率控制在13‰以内，节能减排完成省控目标，主要动态指标继续稳居全省前列。

重点抓好以下八个方面工作：

（一）突出工业扩量提质，大力提升产业竞争力。坚定不移加快新型工业化进程，促进工业总量扩张、结构优化，形成主导产业顶天立地、全民创业铺天盖地、新兴产业惊天动地之势，规模以上工业增加值增长18%以上。

壮大主导产业。围绕“7233”工程，加快建设一批重大项目，工业投资增长20%以上。优化资源配置，做强大企业，做长产业链，做大产业群，力争六大主导产业占规模以上工业销售比重达70%以上。加快建设永通林浆纸一体化、瑞年健康产业园、水晶工业园、中谷产业园，力促冠军建材、瑞年氨基酸、海螺及正鑫水泥二期、麒翔林产品、砀山电动汽车竣工投产，争取罗马陶瓷、金黄庄煤矿、祁南二矿运营，力争钱营孜发电工程开工建设。突出发展纺织鞋服、家居建材产业，加快建设中国现代制鞋产业城、纺织服装产业城、中国宿州（深圳）家居产业园。深入实施白酒五年振兴计划，白酒产业产值突破5亿元。

培育企业梯队。全力扶持实体经济发展，继续实施雏鹰成长、巨人培育和骨干企业技改提升三大行动，推进50户中小微企业上规模，帮扶150户重点调度企业和100户成长性企业，重点实施30个技改项目，加快企业退城进园步伐。着力打造一批主业突出、行业领军的龙头企业，实现一个龙头企业配套一个专业园区、带动一批中小企业，年销售收入超10亿元企业达18家、超20亿元企业达5家，规模以上工业企业力争突破1000户。突出抓好企业生成，大力支持全民创业，出台更具含金量的支持政策，把民营经济发展实效纳入政府考核体系，新发展私营企业2000户、个体工商户1万户。积极争创国家级名牌产品和著名商标。

推进创新转型。重点支持战略性新兴产业发展，开工建设中科院云计算产业园、格微光电二期，加快建设深圳光电产业园，确保符离和官山风电、智慧云计算、润恒光伏发电建成投产，战略性新兴产业产值增长30%。加快自主创新步伐，鼓励企业加大科技信息投入。加快呼叫中心二期建设，科技企业创业服务中心平台交付使用。严格落实节能减排目标责任制，严控“两高一资”项目落地，加快推进结构调整和技术进步，大力发展绿色经济、循环经济和新型低碳产业，确保完成节能减排省控目标。

（二）突出农业增效增收，全面实施美好乡村建设。持续加强“三农”工作，努力在农业增效、农民增收、农村繁荣上下功夫，推动现代农业更好更快发展。

大力发展现代农业。深入开展“两区建设突破年”活动，加快发展粮食、果蔬、畜禽、林木四大主导产业，建设十二大基地，培育三大主体，开展七项试验，加快建设50家现代农业产业联合体，新增2个省级现代农业示范区。深入推进小麦高产攻关和玉米振兴计划，开展高产创建，实现粮食生产稳定增长。积极发展高效农业和生态健康养殖，统筹推进新一轮“菜篮子”工程建设。强力推进农业产业化经营，实施“181”转型倍增计划，农产品加工业产值突破800亿元，超亿元龙头企业突破150家，每个县区培育一个销售收入超20亿元的农业产业化集群。积极开展农业招商，每个县区至少引入2个亿元以上项目。建立农产品质量安全可追溯体系，市县区农产品检验检测中心投入使用。抓好品牌和市场培育，争创名优农产品180个。持续创新现代农业经营体系，规范提升农民专业合作社。深入推进农民收入五年倍增规划，全面落实各种惠农补贴，扩大政策性农业保险范围，加快实施农民就业创业工程，着力增加农民收入。有序推进土地流转，让农民在转包、出租、股份合作中分享增值收益。按照国家新扶贫标准，加大扶贫开发力度，完成整村推进90个村。

不断夯实农业生产基础。继续实施农田水利三年提升工程，全力推进新汴河治理工程，加强大中型灌区续建配套与节水改造，加快小型农田水利重点县建设，完成3条中小河流治理、2座大中型水闸和10座病险小水库除险加固，解决40万农村人口饮水安全问题。建设高标准农田215万亩，完成土地综合治理52万亩。优化水资源配置，加快实施淮北调水、怀洪新河洼地治理工程。健全农业社会化服务体系，加强农业科技创新与推广应用，大力推进农业机械化和信息化，加快打造农产品电子商务平台。

高水平建设美好乡村。高起点编制规划，整合资金资源，深入实施村庄建设、环境整治、产业富民、土地整治和创新管理五大工程，推进首批123个美好乡村建设，突出抓好36个重点示范中心村。加强乡村基础设施建设，广泛开展“三清、四修、五化”，大力实施农村清洁工程和农村危房改造，推进沿河、沿路环境连片整治和景观带打造。创新美好乡村建设管理机制，尊重农民首创精神，完善村民“一事一议”制度。

（三）突出中心城市带动，加速推进新型城镇化。大力实施“园区和城乡建设奋进年”活动，完成投资199亿元，实施四大类221项重点工程，征迁拆违600万平方米。

提升中心城市辐射力。认真落实新一轮城市总体规划，加强控制性规划制定。围绕“132”格局，继续强力推进五大片区建设。主城区重点实施鹏欣水游城广场、三角洲公园改造提升、环城河内环绿地提升、十一中新校区、磬云大市场，继续推进三角洲国际酒店、天赋广场、人民路和拂晓大道两侧改造等项目。城南新区重点实施海关国检服务中心、鞋城商业街、千亩苑安置区等工程。城东新区重点实施区级政务中心、港口路北延及跨新汴河大桥、东外环跨沱河桥等项目。汴北新区重点实施城市规划展示馆、汴北行政服务中心、汴北水厂，铺筑拱辰路、物流大道等主干道路，高标准建设汴北生态大道。高铁新区重点实施洪河路新汴河大桥、学校、自来水厂、污水处理厂及路网建设。加速完善大外环建设，实施两座公跨铁立交桥建设，拉开城市框架，打开片区通道。完善征迁政策机制，推进依法和谐征迁，坚决查处违法建设。强化城市管理，提高精细化、数字化管理水平，改善城市主干道和出入口环境，改善市民交通出行和生活环境。

增强县城和乡镇承载力。加快培育县级中等城市，按照“一县一特”思路，推动四个县城提升功能品位、增强集聚能力。实施“五个一”工程，建设生态大道、市民广场、规划展示馆、高星级酒店，整治城区水系。四个县城建成区均扩区3平方公里，各投入1000万元规划资金、完成100万平方米征迁拆违任务，尽快形成与中心城市互动并进的发展格局。选择5个中心镇，按县城规模建设。加快户籍制度改革，有序推进农业转移人口市民化。

完善城乡基础设施建设。加快建设郑徐客运专线、徐明高速、泗许高速二期、济祁高速、宿淮铁路，扎实推进徐宿阜高速、宿州机场前期工作，力争启动宿淮徐铁路建设。加快国省干线一级公路改造，206国道、310国道、303省道等7条道路升级为一级公路；加快农村公路和场站建设，组织实施县乡公路升级改造150公里、危桥加固改造169座，形成内通外畅的城乡交通网络。实施城乡电网建设改造，新建220千伏变电站1座、110千伏变电站4座。加强城市供水、供气、供热、防洪和信息化建设，加快污水垃圾处理、民防应急等设施配套，实施绿道建设。全面改善城乡生态环境，大力实施森林增长工程，深入推进绿化大行动，主城区新增绿化面积300万平方米，完成14.6万亩植树造林任务。

（四）突出深化改革开放，积极承接产业转移。把改革开放、承接转移作为跨越发展的强大动力，坚定不移加以推进，不断提高对外开放的层次和水平。

积极推进各项改革。推进财税体制改革，巩固扩大“营改增”试点成果。深化预算管理制度改革，强化增收节支，加强财政资金绩效管理。推进收入分配制度改革，促进形成合理有序的收入分配格局。进一步巩固基层医疗卫生机构综合改革成果，基本完成县级公立医院综合改革任务。推进事业单位分类改革，深化文化体制改革，加快国有企业改革步伐。

强力主攻招商引资。加强“5161”项目库建设，形成高质量项目滚动推进的良好局面；争取列入“861”行动计划项目280个，年度投资180亿元。突出招大引强，扎实推进精准招商、产业链招商，实际到位内资突破1000亿元。统筹办好梨花节等会展节庆，认真组织参加徽商大会、中博会等经贸活动，深化与央企、省企、知名民企合作成果。继续推行招商周、领导带头招商、招商任务计划公开等制度，更加注重投资强度考核，完善招商引资考核评价机制。进一步优化发展环境，努力使我市成为全省乃至全国办事效率最高、行政成本最低、投资环境最优、人民群众最满意的地区之一。加快海关、国检设立报批步伐，改进外汇管理与服务，优化外商投资项目资本审批流程，将东艺鞋业纳入保税仓库试点，鼓励支持有条件的企业“走出去”，实际利用外资达4.3亿美元，进出口总额达4.6亿美元。

持续加快园区建设。加快开发区转型升级，提高集聚集约发展水平。放大市经济开发区龙头作用，完成扩区30平方公里，建设亿元以上项目19个，积极创建国家级开发区。提升高新技术产业开发区承载能力，力争入区项目超过10个，着力打造高端高质高效园区。加强宿马现代产业园区建设，引进超亿元企业17家，力争建成全省一流的合作共建园区。县区开发区扩区25平方公里，固定资产投资、招商引资到位资金均增长25%以上。鼓励开展形式多样的园区合作共建。

（五）突出生产生活需求，着力发展现代服务业。扩大消费需求，繁荣城乡市场，提高服务业的支撑力与贡献力。

大力繁荣现代服务业。围绕企业生产经营链，重点发展现代物流、专业市场等基础服务业，科技服务、电子商务等新兴服务业，云计算、软件与互联网等高端服务业，推动智能手机产业园有实质性进展。扩大商贸、家政、养老等生活性服务业，落实节能惠民工程等促进消费政策，提升“万村千乡”市场工程，积极培育新型消费业态，保持消费市场繁荣兴旺，社会消费品零售总额达300亿元。全面启动汴北新区商圈规划工作。加速推进沃尔玛、合百大、红星美凯龙、国购城市综合体、国际汽配城、义乌商贸城等项目建设，新增限额以上企业80家。认真落实国家房地产调控政策，稳步推进房地产业健康发展。

着力拓展金融业。全面落实与各金融机构战略合作协议，扩大信贷投放，全年信贷增量力争达到100亿元。支持地方法人金融机构做大做强，积极培育新的地方金融机构，力争交通银行等股份制银行分支机构入驻，实现徽商银行、淮海村镇银行县域机构全覆盖。深入推进农村合作金融机构改革，启动市区、萧县、砀山联社改制工作。加快开展金融创新，持续深化“金融干部进千企、金融产品进万户、金融服务进千村”活动。大力拓展直接融资渠道，积极推进市建投三期债券、中小企业集合债券发行，推进优质企业上市融资。积极设立非银行金融机构，稳步发展小额贷款公司和产业投资基金。优化金融环境，健全信用担保体系，努力打造省级金融生态优良县。

聚力打造旅游业。围绕打造黄淮海地区旅游目的地，全力抓好皇藏峪5A级、市博物馆和灵璧石文化园4A级景区创建，建成皇藏峪至天门寺环山观光道路，推进磬云山国家地质公园建设、黄河故道综合开发，力争大运河风景区开工，提升蔡洼红色旅游、虞姬文化园、石龙湖湿地公园等景区内涵。积极发展现代农业乡村旅游，实施葡萄酒庄等旅游项目。加快星级饭店创建与提档升级。加大城市形象和旅游景点宣传力度，精心策划包装旅游产品和线路，加快旅游商品认定推介，力争全年接待国内外游客1000万人次，旅游总收入70亿元以上。

（六）突出区域协调发展，强力突破县域经济。把县域经济放在更加突出的战略位置，进一步明晰思路、强化举措、加强帮扶，激发县域经济发展活力。

打造县域特色产业。按照“明确定位，放大优势，错位竞争，组团发展”的原则，鼓励各县区立足自身禀赋，加快特色产业培植，着力构筑具有鲜明县域特色的产业体系。支持桥做大纺织、木材、机械、食品等产业，支持砀山打造以板材、水果加工为基础的农副产品加工业，支持萧县壮大新型建材、绿色合成革、食品和轻化产业，支持灵璧做强纺织、水晶和文化产业，支持泗县发展纺织服装、农副产品加工和文化旅游业，力争每个县区都有一个知名首位产业。

加大县域经济帮扶力度。尽快制定促进县域经济发展措施，加大县区以奖代补力度，增强县区造血功能和自我发展能力。推进土地节约集约利用，加快实施土地整治和增减挂钩，合理配置土地资源，保障县区供地需求。落实扩权强镇政策，大力发展镇域经济、村集体经济，以新型乡村工业园为依托，努力形成一村一品、一镇一业，各县区确保新增3个以上“双超”乡镇。完善县区考核机制，激发县区发展动力，形成奖优罚劣机制。引导萧砀抱团发展，灵泗融合发展，灵互动发展，提升县域经济整体实力。各县区要高点定位，勇于担当，全省争上游，市内争第一，有条件的县区要力争进入全省十强。

（七）突出文化引领方向，努力打造文化强市。解放和发展文化生产力，推动文化大发展大繁荣，不断提高宿州文化软实力。

充分发挥文化引领作用。扎实推进社会主义核心价值体系建设，深入开展社会公德、职业道德、家庭美德和个人品德教育，培育知荣辱、讲正气、作奉献、促和谐的良好风尚。深入推进省级文明城市、文明县城、文明村镇和文明单位创建，继续组织“宿州好人”评选，广泛开展志愿服务，推动学雷锋活动、学习宣传道德模范常态化。健全诚信体系，严惩失信行为。

切实增强公共文化服务能力。深入实施文化惠民工程，提高市博物馆、民间艺术馆开放水平，推动县区图书馆、乡镇文化站提档升级。认真抓好大运河申遗保护工作。规划建设特色文化街区，传承地方特色文化。进一步扩大公共文化产品的有效覆盖面，深化“百戏进百乡”、文化进社区、全民阅读活动，加强文学艺术创作，丰富人民群众精神家园。加强网络管理，开展“扫黄打非”，净化社会风气。

着力提高文化产业竞争力。加大政策扶持力度，构建现代文化产业体系，做大做强灵璧奇石、萧县书画、泗县泗州戏、砀山唢呐、桥马戏五大文化产业。实施重大文化项目带动战略，筹建桥马戏大世界，加速推进文化产业园、星光影视基地、钟馗文化园、书画艺术城、黄河故道风情园等项目建设。大力发展文化创意、演艺娱乐等新兴文化业态，加快建设神游世界动漫产业园、全媒体数字创意产业园、新闻出版印刷产业园，推动文化产业和现代科技深度融合。

（八）突出民计民生改善，全力构建和谐宿州。坚持以人为本、民生为上，着力保障改善民生，统筹发展社会事业，促进公共服务均等化，提高社会和谐度。

完善就业和社会保障体系。实施就业优先战略，加强职业技能培训，突出抓好高校毕业生、农村转移劳动力、城镇困难人员、退伍军人就业工作。促进创业带动就业，加快农民工返乡创业园建设，新建2~3家示范性创业孵化基地，确保新增就业3万人，再就业1.2万人，争创国家级创业型城市。实施九项重点人才工程，统筹推进各类人才队伍建设。全面建设覆盖城乡的社会保障体系，巩固完善城乡居民养老保险制度，“五险”参保人数达到171.8万人次。严格执行最低工资标准，推进工资集体协商，加强劳动保障监察和争议调解仲裁。完善社会救助体系，提高优抚待遇、城乡低保和医疗救助水平。

加快发展各项社会事业。坚持科教优先，加速科技成果转化。大力推进标准化学校建设，继续实施学前教育三年行动计划，加强留守儿童教育，促进教育均衡发展。加快教育园区建设，支持职业技术学院争创省级高职示范院校，力争逸夫师范新校区建成使用，确保皖北卫生职业学院挂牌。加强教师队伍建设。大力发展医疗卫生事业，鼓励社会力量办医，统筹抓好重大传染病防控。认真实施全民健身条例，加快国际网球中心二期建设。严格落实人口计生目标责任制，突出抓好出生人口性别比治理，稳定低生育水平。扎实做好第三次全国经济普查。高度重视档案、外事、残疾人、红十字、防震减灾、侨务和对台工作，发展老龄事业，切实保障妇女和未成年人权益，协调推进供销、气象、烟草、地方志等工作。

维护社会和谐稳定。加强社区和农村警务室建设，强化物业管理指导，引导群众参与社会管理。认真落实安全生产责任制，强化煤矿、非煤矿山、道路交通、建筑施工、消防、危险化学品监管，严防重特大安全事故发生。强化质量技术监督和食品药品安全监管。加强物价监管调控，保持市场基本稳定。以群众工作统领信访工作，深入开展领导干部接访下访活动，建立诉求表达、矛盾排查、舆情研判、社会稳定风险评估机制。完善应急管理机制，创新社会治安防控体系，依法打击违法犯罪活动，不断增强人民群众安全感。全面推进依法治市，扎实开展“六五”普法。依法管理民族宗教事务。支持驻宿部队建设，做好国防动员、人民防空和民兵预备役工作，巩固发展军政军民团结。

巩固提升民生工程。坚持量力而行、普惠民众、滚动发展，在继续实施33项民生工程基础上，再抓好10件惠民实事，让更多的群众享受改革发展成果。

1．实施城乡增绿工程。（新增森林面积2.4万亩，其中“三路一河”造林面积1.2万亩，四个县共完成造林任务1.2万亩。）

2．兴建城市节点游园。（全市兴建16处，其中县区10处。）

3．实施住房保障工程。（新建各类保障房3万套，实施棚户区改造1.64万套。）

4．兴建文体设施。（加快完善县区“三馆一场”，市文化艺术中心、市体育馆竣工。）

5．新建改扩建医疗卫生机构。（加快灵璧和萧县人民医院、泗县和桥中医院、市第一人民医院、市立医院新院区建设，市中医院和市二院综合楼、砀山县人民医院新院区建成使用。）

6．新建“四有”标准示范社区12个。

7．新建19所公办幼儿园和1所九年一贯制学校。

8．继续推进主城区清水工程，改造疏浚河道17.1公里。

9．继续推进新汴河城区段治理，完成3.9公里主景观带建设。

10．完成主城区自来水一户一表改造5000户，3万户居民用上暖气。

四、进一步加强政府自身建设

实现宏伟蓝图，使命崇高，责任重大。我们必须主动作为，全面加强政府自身建设，全力打造为民、效能、法治、廉洁的服务型政府。

全力打造履职尽责、服务至上的为民政府，不断增强发展推动力。践行为民宗旨，严格执行中央八项规定和省30条要求，改进工作作风，密切联系群众，始终与人民心心相印、与人民同甘共苦、与人民团结奋斗。改进调查研究，多到困难和矛盾集中、群众意见多的地方去，真正问政于民、问需于民、问计于民，使政府工作更加契合民情民意。坚定理想信念，保持公仆情怀，夙夜在公，勤勉工作，以实干创造实绩、凭实绩赢得民心。

全力打造勇于担当、务实创新的效能政府，不断增强行政执行力。坚持不懈抓好效能建设，持之以恒推行“六要六不要”，大力消除“懒慢推假冷脏”等现象。点燃干事激情，强化责任落实，坚持密集调度，严格考核奖惩，推动政府工作持续提速提效。继续推进“四个一”工程，进一步清理行政审批事项，提供更加优质高效的政务服务。加强政府工作创新，深化政务公开，积极推进网上审批、网络问政、微博问政。

全力打造科学规范、阳光透明的法治政府，不断增强政府公信力。健全重大问题集体决策、风险评估、专家咨询、公示听证、跟踪问效等机制，提高政府决策科学化、民主化水平。坚持依法行政，推进行政复议试点工作，规范行政执法自由裁量权，促进规范执法、公正执法、文明执法。自觉接受人大法律监督和政协民主监督，切实办好人大议案、代表建议和政协委员提案。主动接受群众和舆论监督，提高政府工作透明度和群众满意度。

全力打造勤勉清廉、风清气正的廉洁政府，不断增强制度约束力。严格执行党风廉政建设责任制，加强廉政风险防控，强化审计监督，更加科学有效地防治腐败，做到干部清正、政府清廉、政治清明。坚决纠正损害群众利益的不正之风，着力解决发生在群众身边的腐败案件。厉行勤俭节约，严格执行住房、车辆配备等有关工作和生活待遇的规定。力戒形式主义，下决心改进文风会风。加强干部管理考核，努力建设一支有激情、能干事的高素质公务员队伍。

各位代表！新的征程已经开启，新的目标催人奋进。让我们紧密团结在以习近平同志为总书记的党中央周围，在市委的坚强领导下，进一步解放思想、锐意创新，励精图治、扎实工作，为实现“四个宿州”宏伟目标、全面建成小康社会而努力奋斗！
